# Socio-demographic and maternal predictors of adherence to 24-hour movement guidelines in Singaporean children

**DOI:** 10.1186/s12966-019-0834-1

**Published:** 2019-08-22

**Authors:** Bozhi Chen, Jonathan Y. Bernard, Natarajan Padmapriya, Jiali Yao, Claire Goh, Kok Hian Tan, Fabian Yap, Yap-Seng Chong, Lynette Shek, Keith M. Godfrey, Shiao-Yng Chan, Johan G. Eriksson, Falk Müller-Riemenschneider

**Affiliations:** 1 Saw Swee Hock School of Public Health, National University of Singapore, Tahir Foundation Building (Block MD1), 12 Science Drive 2, #09-01v, Singapore, 117549 Singapore; 2 Inserm, Centre for Research in Epidemiology and StatisticS (CRESS), Research team on Early life Origins of Health (EAROH), Villejuif, France; 3 Agency for Science Technology and Research (A*STAR), Singapore Institute for Clinical Sciences (SICS), Singapore, Singapore; 4 Department of Obstetrics & Gynaecology, Yong Loo Lin School of Medicine, National University of Singapore, National University Health System, Singapore, Singapore; 5 Department of Maternal Fetal Medicine, KK Women’s and Children’s Hospital, Singapore, Singapore; 6 Duke-NUS Medical School, Singapore, Singapore; 7 Department of Paediatrics, KK Women’s and Children’s Hospital, Singapore, Singapore; 8 Lee Kong Chian School of Medicine, Nanyang Technological University, Singapore, Singapore; 9 Department of Paediatrics, Yong Loo Lin School of Medicine, National University of Singapore, Singapore, Singapore; 10 Division of Paediatric Allergy, Immunology & Rheumatology, Khoo Teck Puat - National University Children’s Medical Institute, National University Health System, Singapore, Singapore; 11 Medical Research Council Lifecourse Epidemiology Unit, University of Southampton, Southampton, UK; 12 NIHR Southampton Biomedical Research Centre, University of Southampton and University Hospital Southampton NHS Foundation Trust, Southampton, UK; 13 Institute for Social Medicine, Epidemiology and Health Economics, Charite University Medical Centre, Berlin, Germany

## Abstract

**Purpose:**

Integrated 24-Hour Movement Guidelines provide specific recommendations on screen viewing (SV), moderate-to-vigorous physical activity (MVPA) and sleep to improve health of children and youth. However, few studies have examined whether these guidelines are met in young children, particularly in Asia. We evaluated adherence to integrated and individual guidelines and its predictors in 5.5-year-old Singaporean children.

**Methods:**

Growing Up in Singapore towards Healthy Outcomes (GUSTO) is a mother-offspring birth cohort study. At age 5.5 years, child SV was reported by parents. Movement behaviours (MBs) were measured continuously using wrist-worn accelerometers over 7 consecutive days and nights. For accelerometer data including ≥3 days with ≥16 h/day we estimated mean (±SD) daily MVPA, SV and nighttime sleep duration across the week. Adherence to integrated (Canadian/Australian) guidelines was defined as meeting all individual guidelines: ≥60 min of MVPA/day, ≤2 h of screen time/day, and 9–11 h of sleep/night. Socio-demographic and maternal predictors collected at pregnancy enrolment and at 26–28 weeks’ gestation were examined by multivariable logistic regression.

**Results:**

Of 864 children followed up age 5.5 years, 547 (63.3%) had both valid ActiGraph and questionnaire data (51.7% boys and 58.3% Chinese ethnicity). Children averaged 101.9 (± 88.7) min/day SV, 67.3 (± 23.7) min/day MVPA and 480.6 (± 57.2) min/night sleep. Few children met integrated guidelines. Specifically, the proportions of children who met none, SV, MVPA, sleep and integrated guidelines were 11.2, 70.2, 59.6, 13.7 and 5.5%, respectively. Multivariable analysis showed that maternal activity and television (TV) viewing were associated with meeting integrated guidelines (insufficiently vs. highly active (OR [95% CI]): 0.11 [0.01, 0.95]; 2–3 vs. ≥ 3 h TV: 3.52 [1.02, 12.22]). Examining higher adherence to individual guidelines, Chinese ethnicity, younger maternal age and lower maternal TV and sleep time were associated with greater SV; male sex, Malay ethnicity, higher birth order and higher maternal activity level were associated with greater MVPA; and older maternal age was associated with adherence to sleep guideline.

**Conclusions:**

Beyond individual behaviours, consideration of the full spectrum of MBs may be important to improve children’s health. However, few Singaporean children adhere to integrated 24-h movement guidelines. Maternal behaviours as early as during pregnancy could be important targets for future interventions aiming to promote these MBs in children.

**Electronic supplementary material:**

The online version of this article (10.1186/s12966-019-0834-1) contains supplementary material, which is available to authorized users.

## Introduction

Children’s physical activity (PA), screen-based sedentary behaviour (SB) (also known as screen viewing (SV) and sleep are independently associated with health and well-being. Studies have consistently shown that higher levels of PA are associated with improved motor development, healthier cardio-metabolic function and better psychosocial health [1, 2]. Greater SV among young children, on the other hand, has been associated with higher cardio-metabolic risk, unfavorable body composition and behavioural conduct, and lower self-esteem [3, 4]. Insufficient sleep duration has been associated with higher adiposity risk, decreased emotional regulation, less successful academic achievements and worse quality of life [5–7]. Further, the combination of these behaviours affect a number of important health indicators [8, 9]. These behavioural patterns establish in early childhood and persist into adulthood [10]. To promote health and well-being in children and adults it is therefore essential to understand and improve the balance between these behaviours at young age.

Physical activity, SB and sleep represent the movement spectrum across the entire day and have been referred to as “movement behaviour (MB)” [11]. Consequently, it is flawed to view them individually because time spent in one behaviour is co-dependent with/on the other behaviours during the remaining time of the day. Since 2016, Canada [11, 12], Australia [13, 14], South Africa [15] and WHO [16] have developed integrated movement guidelines for children aged 0–17 years, taking into consideration all behaviours that make up an entire day. While most of these guidelines focus only on children up to 5 years, Canada and Australia also provide recommendations for children aged 5–17 years. In the Canadian and Australian 24-Hour Movement Guidelines for children and youth, for instance, recommendations include sufficient PA (e.g. ≥60 min/day of MVPA), together with limited SV (≤2 h/day) and adequate sleep duration (e.g. 9–11 h/night for children aged 5–13).

Meeting the integrated guidelines appears to have a greater positive impact on a child’s cognition [17], physical and psychological heath [18, 19], than meeting only one guideline or not meeting any guideline. As reported by the few available studies, adherence to integrated guidelines ranged from 11.9 to 30% in children [20–23]. However, these studies have largely been conducted in Canada or Australia and to the best of our knowledge, no study from Asia has yet been published. Existing studies on isolated MBs in Asia have reported a high prevalence of physical inactivity [24], excessive time spend on screen devices [4, 25], and an average sleep duration that is shorter compared to children from the US or Europe [26]. Therefore, adherence to integrated guidelines may be even lower among Asian children.

Studies have explored potential predictors of single MB in young children [26–28]. As suggested by such studies, some socio-demographic (e.g. child’s age and sex) and maternal factors (e.g. maternal age and activities) were associated with children’s individual MB. Previous research has also shown strong links between pre-pregnancy behaviours and subsequent child health [29]; however, studies investigating maternal behavioural factors generally used cross-sectional study designs, and none explored maternal pre-pregnancy behaviours which could present a window of opportunity for health promotion activities to influence the manifestation of health behaviours in early childhood. Maternal behaviours during early pregnancy have also been reported to be associated with maternal physical and mental health [30]. Thus, maternal pre-pregnancy behaviours may be predictors of children’s heath, through their associations with children’s health behaviours during early childhood. To our knowledge, there is no study that investigated the association between these factors and adherence to integrated guidelines in young children.

Utilizing data from the Growing Up in Singapore Towards Healthy Outcomes (CUSTO) mother offspring study we aimed to address existing research gaps. Specifically, we aimed a) to investigate the proportion of children meeting individual (MVPA, SV, and sleep) and integrated movement guidelines at 5.5 years in a multi-ethnic Asian population, and b) to identify the socio-demographic and maternal pre-pregnancy factors associated with adherence to the integrated and individual guidelines.

## Methods

### Study population

This study was part of the Growing Up in Singapore Towards Healthy Outcomes (GUSTO) study, a mother-offspring birth cohort study examining early-life factors that affect long-term health and development of children. Study design and detailed protocols have been described elsewhere [31]. Briefly, from June 2009 to September 2010, pregnant women were recruited during their first ultrasound scan visit to two public maternity units in Singapore (KK Women’s and Children’s Hospital and National University Hospital). Inclusion criteria were: 1) being Singaporean citizens or permanent residents with homogeneous Chinese, Malay or India ethnicity; 2) intending to deliver in one of the two above-mentioned maternity units and remain in Singapore for the following 5 years. Of the 1247 pregnant women recruited, 1171 singleton newborns were included and followed up regularly. Written informed consent was obtained from all participants at enrolment. This study was approved by the National Healthcare Group Domain Specific Review Board and the SingHealth Centralised Institutional Review Board.

### Data collection

As part of an interviewer-administered questionnaire, socio-demographic information was obtained at enrolment, including ethnicity (Chinese, Malay, India), maternal highest level of education (primary or secondary, post-secondary, university), marital status (single, married), monthly household income (< 4000, 4000–5999, ≥6000 Singapore dollars). Information on maternal age at delivery (< 30, 30–35, ≥35 years) and the offspring’s date of birth, sex and birth order (first-, second-or later-born) was extracted from medical records. Self-reported pre-pregnancy weight and height collected at the 26–28 weeks gestational visit were used to calculate pre-pregnancy BMI (kg/m^2^). BMI was categorized as underweight, normal weight, overweight and obese using cut-offs for Asian populations (< 18.5, 18.5–23, 23–27.5, ≥27.5 kg/m^2^) [32]. Pre-pregnancy behavioural information (i.e. PA, television (TV)-viewing, sleep) was obtained through an interviewer-administered questionnaire at the 26–28-week visit. Six items assessing frequency and duration of PA at different intensities were adopted from a structured questionnaire [33] to determine the total time spent at each intensity level of maternal PA before pregnancy; PA was then categorized as insufficiently, sufficiently or highly active, as detailed previously [34]. Energy expended on PA at each intensity was derived by multiplying weekly total minutes and its corresponding metabolic equivalent task (MET) value (3.3, 4.0 and 8.0 for light, moderate and vigorous intensity respectively [35]). Total energy expenditure on PA per week was calculated by summing energy expenditure on PA at all intensities, then categorized into insufficiently active (< 600 MET-minutes/week), sufficiently active (600–3000 MET- minutes/week), and highly active (≥3000 MET- minutes/week). In addition to PA assessment, maternal TV-viewing time and total sleep duration during a 24-h period before pregnancy were also assessed as part of the questionnaire at 26–28-week visit. TV-viewing time was categorized into three levels (< 2, 2–3, ≥ 3 h), consistent with literature suggesting that children whose parents watched TV more than 2 h/day were more likely to have greater SV [36]. Our categorization of daily total sleep duration (< 7, 7–9, ≥ 9 h) was based on the recommended sleep range (i.e. 7–9 h/day) for adults [37].

### Accelerometer-measured physical activity (PA) and sleep duration among children

PA was measured objectively using triaxial accelerometers (ActiGraph™ wGT3X-BT) which were set to record raw acceleration data. At the age 5.5 years study visit, researchers attached an accelerometer on each child’s non-dominant wrist with a non-removable strap. Accelerometers were set to start at midnight on the following day after the home visit; no end date or time were set. Children were asked to wear the accelerometer for 7 days and nights and remove it on the 8th day; allowing to record 24-h activities throughout a week.

Data were collected at a rate of 80 Hz and downloaded in raw format (GT3X) with the Actilife software (version 6.13). Raw data were processed in R using the GGIR package (version 1.6–0) [38]. Raw triaxial accelerometer signals were auto-calibrated [39] and converted into gravity-corrected vector magnitude units, termed the Euclidean norm minus one (ENMO) [38]. Accelerometer wear time inclusion criteria were a minimum of 16 h/day for at least 3 days. Non-wear time was estimated based on the standard deviation and value range of each accelerometer axis, using a 60-min window with 15-min increments. For each 15-min period detected as non-wear time over the valid wearing days, the invalid data were imputed using the mean value of valid data at same time points on other days [40]. Sleep duration was estimated using the method by van Hees et al. as part of GGIR processing [41]. We used the acceleration intensity thresholds (m*g*) to classify activity during waking time into SB (ENMO≤35.0 m*g*), light PA (LPA, ENMO 35.0–200.0 m*g*) and MVPA (ENMO> 200.0 m*g*), which were identified by Hildebrand et al.’s prediction eqs. [42].

### Proxy-reported screen viewing (SV) among children

During the study visit at age 5.5 years, parents were asked to report the amount of time their child spent watching/using TV/DVD, computers, mobile devices and video game consoles on weekdays, Saturday and Sunday (see Additional file 1: Table S1 for the items). The items were from the preschool-age physical activity questionnaire (Pre-PAQ), a questionnaire validated in preschool-aged children [43]. Pre-PAQ was modified slightly by including one additional question about time spent on mobile devices, to make it more applicable to the current Singaporean context. Daily device-specific SV time was calculated as follows: ((Weekdays × 5) + Saturday + Sunday) / 7. Total SV was calculated by adding up the daily SV time of each device.

### Statistical analysis

Means ± standard deviations (SD), medians (interquartile range (IQR)) for continuous, frequencies and percentages for categorical variables were estimated. Pearson’s χ2 test was conducted to compare the socio-demographic characteristics (i.e. child’s sex, ethnicity, birth order; household income and maternal education) of children who were included with those not included in the further analyses. Our study applies the Canadian and Australian 24-Hour Movement Guidelines for children and youth [11, 14], because the study sample fits better in their target age group 5–17 years. We calculated the proportions of children meeting the weekly averaged MVPA (≥60 min/day), SV (≤2 h/day), sleep (9–11 h/night) guideline [11], and their combination to determine the proportion of children meeting the integrated guidelines.

The probabilities that a child met the integrated guidelines and its individual components were modelled using univariate and multivariable logistic regression. In the multivariable model, we examined the following correlates: child’s age (in years), sex, ethnicity, birth order, household income, maternal education, maternal pre-pregnancy BMI, maternal age at delivery, maternal PA, TV and sleep before pregnancy. All statistical analyses were conducted using R version 3.4.3.

## Results

**Fig. 1:**
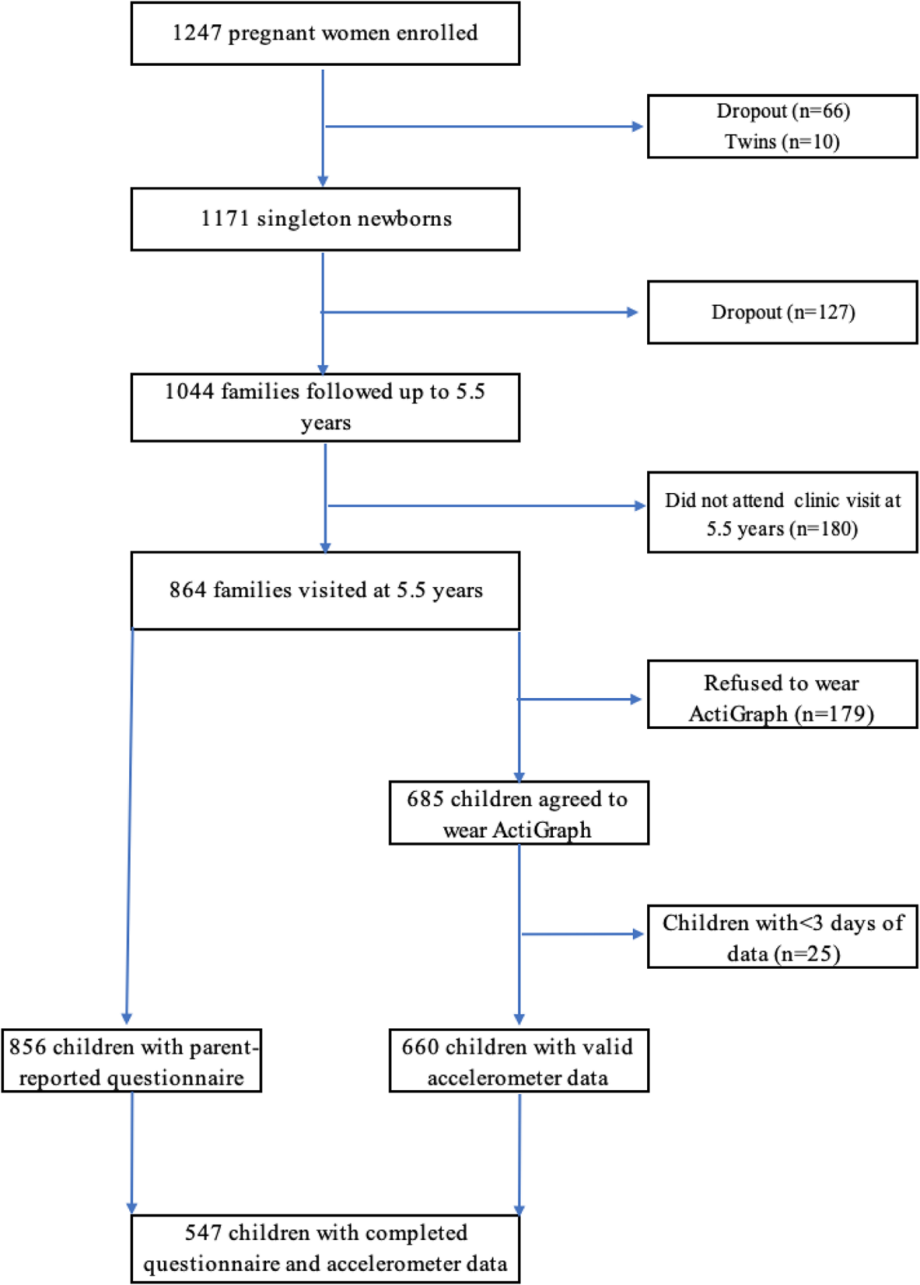
Flow diagram of the GUSTO study participants followed up to 5.5 year

Of 864 children who attended the clinic visit at age 5.5 years, 547 (63.3%) had both parent-reported SV and valid accelerometer data (Fig. 1). No significant difference (*P* > 0.05 for all variables) was observed between children included in the study and those not included (data not shown). Detailed child and maternal characteristics are presented in Table 1. Briefly, the mean age was 5.5 (± 0.1) years, 51.7% were boys and 58.3% were of Chinese ethnicity, with the remainder being of Malay (24.1%) or Indian (17.6%) ethnicity.

**Table 1:** Characteristics of 5.5-year-old children and their mother in the GUSTO study (N = 547)

	N	Mean ± SD or %
Child’s age (year)	547	5.5 ± 0.1
Sex		
Boy	283	51.7
Girl	264	48.3
Ethnicity		
Chinese	319	58.3
Malay	132	24.1
Indian	96	17.6
Birth order		
First-born	244	44.6
Second-or later-born	303	55.4
Household incomes (SGD/month)		
< 4000	237	46.1
4000–5999	117	22.8
≥ 6000	160	31.1
missing, *n*	33	
Maternal education		
University	187	34.4
Post-secondary	190	34.9
Primary or secondary	167	30.7
missing, *n*	3	
Pre-pregnancy weight status		
Underweight/normal	340	62.2
Overweight	133	24.3
Obese	74	13.5
Maternal age at delivery		
< 30	223	40.8
30–35	171	31.3
≥ 35	153	28.0
Maternal physical activity before pregnancy		
Insufficiently active	99	18.4
Sufficiently active	296	54.9
Highly active	144	26.7
missing, *n*	8	
Maternal daily television-viewing time before pregnancy		
< 2 h	260	48.1
2–3 h	133	24.6
≥ 3 h	147	27.2
missing, *n*	7	
Maternal daily total sleep time before pregnancy		
< 7 h	71	13.1
7–9 h	335	62.0
≥ 9 h	134	24.8
missing, *n*	7	

**Fig. 2:**
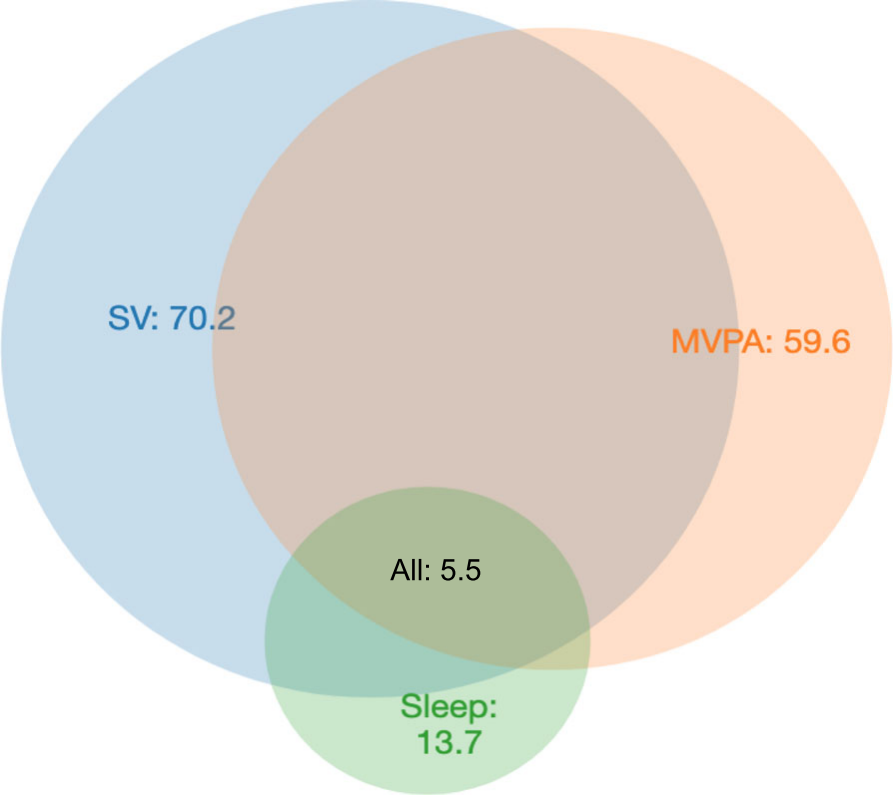
Venn diagram of the proportions (%) of participants meeting screen viewing (SV), moderate-to-vigorous physical activity (MVPA), sleep duration recommendations and combinations of these recommendations (*N* = 547)

Table 2 shows the descriptive characteristics of parent-reported SV, accelerometer-measured PA and sleep, and the proportions of children meeting the individual and integrated guidelines. On average, children spent 101.9 ± minutes on total SV (TV: 49.4 ± 49.4 min; mobile devices: 34.8 ± 47.3), engaged in 431.9 ± 67.3 min/day of PA (including 67.3 ± 23.7 min of MVPA), and spent 480.6 ± 57.2 min sleeping at night. Figure 2 illustrates the proportions of the children meeting the SV, MVPA, and sleep guidelines, as well as the combination of these: 5.5% of the children met the integrated guidelines. This proportion ranged from 2.0 to 8.3% across different subgroups. Children of an insufficiently active mother before pregnancy had the lowest adherence to integrated guidelines, whereas Indian children had the highest. In terms of meeting individual guidelines, 70.2% of the children met SV guideline, with highest proportion (81.8%) in children of mothers educated to university level and lowest (53.0%) in Malay children; 59.6% met the MVPA guideline, with the highest proportion (67.8%) in boys and lowest (45.5%) in children of an insufficiently active mother before pregnancy; 13.7% met sleep guidelines with the highest proportion (20.3%) in children of mothers older than 35 years at delivery and the lowest (7.0%) in children of mothers sleeping less than 7 h before pregnancy. Overall, 11.2% did not meet any guidelines, with the highest proportion (17.2%) in children of an insufficiently active mother before pregnancy and the lowest (3.8%) in children from families with a monthly household income more than 6000 SGD. Details of the proportions of children meeting no guidelines, individual and integrated guidelines by each socio-demographic or maternal factor are summarised in Additional file 1: Table S2.

**Table 2:** Daily time (min/day) spent in self-reported screen viewing and accelerometer-measured physical activity and sleep at age 5.5 y in the GUSTO cohort (N = 547)

	Mean ± SD	Median (IQR)
Screen viewing (total)	101.9 ± 88.7	77.1 (42.1–140.7)
Television	49.4 ± 49.4	37.1 (9.3–64.3)
Computer	12.6 ± 28.5	0.0 (0.0–17.1)
Mobile devices	34.8 ± 47.3	17.1 (0.0–51.4)
Game consoles	3.6 ± 14.7	0.0 (0.0–0.0)
Physical activity (total)	431.9 ± 67.3	437.9 (388.9–477.4)
Light intensity	364.6 ± 57.0	371.3 (324.6–401.0)
Moderate-to-vigorous intensity	67.3 ± 23.7	64.8 (49.8–81.6)
Sleep duration (total)	538.8 ± 58.1	542.0 (501.3–578.8)
Night-time sleep	480.6 ± 57.2	483.6 (442.2–524.0)
Daytime sleep	58.3 ± 29.2	56.0 (37.3–74.7)

Abbreviation: *SD* (standard deviation); *IQR* interquartile range)

Table 3 shows the adjusted odds ratios for the associations of socio-demographic and maternal factors with adherence to the integrated and individual guidelines at age 5.5 years. Only higher maternal PA level (sufficiently active: 0.67 [0.29, 1.56]; insufficiently active: 0.11 [0.01, 0.95]) and lower maternal TV time (< 2 h TV: 1.58 [0.47, 5.28]; 2–3: 3.52 [1.02, 12.22]) before pregnancy were significant predictors of higher adherence to integrated guidelines. In addition, compared to Chinese children, Malay children were less likely to meet SV (0.33 [0.19, 0.58]) and more likely to meet MVPA guidelines (1.76 [1.03, 3.00]). Younger maternal age at delivery was associated with higher adherence to SV (< 30 years: 1.73 [1.00, 2.97]; 30–35: 1.23 [0.72, 2.09]) but lower adherence to sleep guidelines (< 30 years: 0.63 [0.33, 1.2]; 30–35: 0.41 [0.20, 0.84]). Children of an insufficiently active mother before pregnancy were less likely to meet the MVPA guideline (0.44 [0.25, 0.78]); those whose mothers watched less than 2 h TV per day before pregnancy were more likely to meet SV guidelines (1.73 [1.05, 2.84]). Maternal pre-pregnancy sleep time was significantly associated with children’s adherence to SV (< 7 h/night: 2.15 [1.05, 4.42]; 7–9: 1.49 [0.92, 2.41]). Other predictors significantly associated with higher adherence to MVPA were: being a boy, or a second- or later-born child.

**Table 3:** Adjusted associations of socio-demographic and maternal factors with adherence to screen viewing (SV), moderate-to-vigorous physical activity (MVPA), sleep duration recommendations and combinations of these recommendations

	All	SV	MVPA	Sleep
	OR (95% CI)	OR (95% CI)	OR (95% CI)	OR (95% CI)
Sex (ref: girl)				
Boy	0.81 (0.36, 1.80)	1.20 (0.80, 1.80)	**1.94 (1.33, 2.81)**	0.94 (0.56, 1.59)
Ethnicity (ref: Chinese)				
Malay	1.11 (0.34, 3.65)	**0.33 (0.19, 0.58)**	**1.76 (1.03, 3.00)**	1.85 (0.90, 3.81)
Indian	1.54 (0.52, 4.55)	0.90 (0.49, 1.66)	1.15 (0.66, 1.99)	1.55 (0.73, 3.32)
Birth order(ref: first-born)				
Second-or later-born	2.16 (0.84, 5.58)	0.76 (0.48, 1.19)	**1.59 (1.05, 2.40)**	1.42 (0.79, 2.57)
Maternal education (ref: university)				
Primary or secondary	0.99 (0.28, 3.47)	0.82 (0.42, 1.62)	0.67 (0.36, 1.25)	1.52 (0.64, 3.60)
Post-secondary	1.08 (0.35, 3.30)	0.64 (0.35, 1.16)	1.02 (0.60, 1.74)	1.02 (0.46, 2.24)
Household incomes (ref: < 4000 SGD/month)				
4000–5999	0.62 (0.18, 2.15)	0.83 (0.48, 1.42)	1.49 (0.89, 2.50)	0.69 (0.31, 1.53)
≥ 6000	1.38 (0.41, 4.58)	1.25 (0.64, 2.43)	1.62 (0.89, 2.97)	1.61 (0.70, 3.70)
Pre-pregnancy weight status (ref: underweight/normal)				
Overweight	0.99 (0.37, 2.67)	0.86 (0.52, 1.41)	1.02 (0.64, 1.63)	1.45 (0.77, 2.73)
Obese	0.55 (0.14, 2.17)	0.58 (0.32, 1.06)	0.83 (0.46, 1.50)	1.13 (0.51, 2.51)
Maternal age at delivery (ref: ≥35)				
< 30	0.78 (0.29, 2.14)	**1.73 (1.00, 2.97)**	1.33 (0.81, 2.17)	0.63 (0.33, 1.20)
30–35	0.43 (0.15, 1.26)	1.23 (0.72, 2.09)	1.18 (0.73, 1.92)	**0.41 (0.20, 0.84)**
Maternal physical activity before pregnancy (ref: highly active)				
Sufficiently active	0.67 (0.29, 1.56)	1.10 (0.68, 1.79)	0.79 (0.50, 1.24)	1.01 (0.55, 1.88)
Insufficiently active	**0.11 (0.01, 0.95)**	0.79 (0.43, 1.47)	**0.44 (0.25, 0.78)**	1.24 (0.56, 2.75)
Maternal daily television-viewing time before pregnancy (ref: ≥ 3 h)				
< 2 h	1.58 (0.47, 5.28)	**1.73 (1.05, 2.84)**	0.83 (0.51, 1.33)	1.22 (0.63, 2.39)
2–3 h	**3.52 (1.02, 12.22)**	1.03 (0.60, 1.78)	0.97 (0.57, 1.66)	1.32 (0.62, 2.79)
Maternal daily sleep time before pregnancy (ref: ≥ 9 h/night)				
< 7 h	0.37 (0.07, 1.98)	**2.15 (1.05, 4.42)**	0.71 (0.37, 1.37)	0.43 (0.15, 1.29)
7–9 h	0.90 (0.35, 2.33)	1.49 (0.92, 2.41)	0.76 (0.48, 1.20)	1.19 (0.64, 2.23)

Note: logistic regression was conducted to test the association between predictors and meeting guidelines; all predictors were included in each model; bold: significant associations (*p* < 0.05)

## Discussion

In our multi-ethnic Singaporean mother-offspring cohort, we found that only 5.5% of children aged 5.5 years met integrated 24-Hour Movement Guidelines and 11.2% of the children met none of the movement guidelines. Accordingly, a substantial proportion of children did not meet individual guidelines, which was particularly frequent with regard to sleep. Pre-pregnancy maternal PA and TV time were the only significant predictors of adherence to integrated guidelines at 5.5 years of age; other socio-demographic and maternal predictors were associated with individual guidelines, demonstrating inconsistent patterns.

A previous study including children from different continents, demonstrated that meeting integrated guidelines was associated with the lowest odds ratio of obesity when compared with meeting individual or no guidelines [19]. Similar findings have also been reported in a study of American children [44]. Despite this evidence for the importance of considering all MBs, to our knowledge no study has examined the adherence to 24-Hour Movement Guidelines among children at similar age as in our study. Similar to our findings, Roberts et al. [21] reported that among Canadian children aged 5–11 years, 70.6 and 46.8% met SV and MVPA guidelines, respectively. However, much higher adherence to integrated (29.6%) and sleep guidelines (82.6%) was reported in their study. The low adherence in the current study is consistent with previous studies investigating the adherence to the Canadian 24-Hour Movement Guidelines for Children and Youth in older children. For instance, Walsh et al. [17] reported that 5.0% of 8–10 year olds met the integrated guidelines in the U.S. In another large 12-country study (including 8 Western, 2 Asian and 2 African countries) of 6128 children aged 9–11 years, 7.2% of the children met integrated guidelines, with the lowest adherence (1.5%) in China [19]. Possible reasons for different behavioural patterns of preschool-aged children in Asia could be a stronger academic focus in society and a tendency for longer preschool hours among children in Asia [45]. Inadequate play opportunities for the enhancement of PA are likely to occur inside preschool and additional enrichment classes may leave little time for children to play outside the school environment [46]. Although a relatively high proportion of children met SV guideline in our sample, it is still concerning that almost 30% spent excessive time on SV. The proportion of children meeting sleep recommendations in this study was particularly small and children slept less than 9 h per night on average. These findings are consistent with previous evidence that young Singaporean children spent an average of only 8.8 h on night-time sleep [47]. Studies among similar-aged children from other high-income countries/regions in Asia have reported similar observations [48, 49]. These estimates are much lower than those in Western countries, such as Australia [23], Canada [22], the U.S. [50], or European countries [51]. The differences of night-time sleep duration between these Western studies and our current study may be partly due to discrepancies in daytime napping behaviour across cultures [52], and nap duration has been shown to negatively correlate with night-time sleep duration in young children [53]. In Singapore and many other Asian countries and regions, napping is an important part of preschool daily schedule and children at this age are known to have mandatory afternoon naps at preschools [47]; on the contrary, this may not be possible for children in most Western countries due to a shorter preschool day. Children in the current study napped for around 1 h/day on average, whereas very few children of similar age have been reported to nap in Western countries [51, 54]. Considering napping in addition to nigh-time sleep increased the proportion of children meeting the sleep and integrated guidelines in our study considerably. However, the proportions of children meeting sleep guidelines remained lower than in Western preschool children. These results suggest that the current movement guidelines developed in Western countries may not be directly applicable to Asia, because they do not take these specific cultural aspects of sleeping and napping behaviour into consideration. When developing integrated guidelines for Singapore or Asia it may therefore be necessary to pay particular attention to the element of sleep and napping behaviour.

Understanding factors that contribute to low adherence towards integrated movement guidelines is necessary for health promotion efforts to control the childhood obesity epidemics [55]. In the current study, only higher pre-pregnancy maternal PA and TV time less than 3 h/day were associated with higher adherence to integrated guidelines. Maternal PA and TV time were also associated with children’s adherence to MVPA and SV, respectively. These findings expand on previous studies reporting cross-sectional associations between maternal and children’s behaviours [56–58], re-iterating the importance of targeting maternal behavioural factors in order to promote children’s health behaviours. Previous research indicated that maternal sleep/wake patterns affect the sleep/wake patterns of their children [59], but we did not find a link between maternal sleep before pregnancy with children’s adherence to sleep guideline. However, we cannot rule out the possibility that current maternal sleep may be important. Previous systematic reviews [59, 60] have highlighted the importance of engaging and supporting parents in the promotion of children’s healthy behaviours, however, health promotion efforts have tended to focus on school settings. A systematic review of existing intervention studies in Asia, reported that all but one study occurred in a school-based setting and all targeted children at school age or above [61]. In light of our findings and previous evidence [25] with regards to the impact of detrimental parental behaviours (e.g. excessive SV and limited PA levels), it may be necessary for future interventions to also involve parents, with additional efforts on parental behaviours. Especially during early pregnancy women may be open to health promoting messages and activities which are beneficial for both themselves and their child in the future [62].

The lack of other consistent predictors of adherence to integrated guidelines is likely due to the fact that predictors of individual guidelines were highly variable and behaviour-specific. For instance, younger maternal age was significantly associated with higher adherence to SV but a lower adherence to sleep guidelines. Children of mothers younger than 35 years were more likely to sleep 9–11 h, which confirms previous evidence that children with younger mothers tend to sleep more [63]. Although maternal pre-pregnancy weight was not a predictor of children’s behaviours at 5.5 years, previous cross-sectional studies have found associations between maternal weight and children’s PA and SB in Western populations [64]. Apart from maternal predictors, girls were less likely to meet MVPA guideline at 5.5 years in this study. Our finding is consistent with other studies [27, 65, 66] suggesting sex differences in PA may be observed early in life, warranting early intervention to promote PA in girls. Contrary to previous studies [67–69], we did not observe significant sex differences in SV or sleep guideline adherence in our sample. As studies [70, 71] have suggested that boys spend more time playing screen devices after age 5 years, sex differences in SV may emerge as they age. In addition to age, our study reported that Malay children were more likely to adhere to MVPA but less likely to SV guideline than Chinese. Although the relationship between ethnicity and childhood SV may be country- and context-specific, the available literature suggests that children from ethnic majorities spend less time on screen devices [18, 24–26].

This is the first study to assess adherence to MVPA, SV and sleep of the 24-h movement guidelines and to identify the main predictors of adherence to these guidelines in an Asian population. We assessed a wide range of socio-demographic and maternal predictors and used objectively-measured PA and sleep time. In addition to providing locally specific information that may help develop national health promotion strategies, this study provides information that will enable international comparisons of adherence to integrated movement guidelines among young children, and their predictors. For instance, our findings may be able to inform the development of national report card on PA for children as part of global initiatives in promoting PA [72]. Therefore, our results may provide important information for both local- and international-level efforts to promote children’s healthy living.

However, we acknowledge certain limitations of our study. First, children’s time spent on screen devices was reported by parents, which could be underreported when children were at childcare centres or parents were not at home. However, these items are derived from a validated questionnaire and have been used in other studies [73, 74]. Second, pre-pregnancy behavioural data (including PA, TV and sleep duration) were limited to mothers’ self-reports with potential recall bias. Thirdly, we did not capture information on current maternal behaviours. Hence, there might be a chance that pre-pregnancy behaviours was just a reflection of their current behaviours. Fourthly, the current 24-Hour Movement Guidelines do not account for napping behaviour for children aged 5 years and above, which is common among Asian children. This may result in an underestimation of the proportion of children meeting sleep and integrated guidelines. Integrated guidelines for Singapore or other Asian countries may therefore have to include recommendations on the total sleep duration in a 24-h period rather than only night time sleep. Finally, although our sample is very similar to the entire GUSTO sample, the cohort does not represent the entire Singaporean population. For instance, Malay and Indian families were overrepresented purposely at inclusion; recruited mothers were also less likely to hold a university degree than the women from the general population of the same age range [31]. Generalizing our results should therefore be made with caution.

## Conclusions

Only about 1 in 20 Singapore preschool-aged children met integrated movement guidelines and about 1 in 10 failed to meet any guideline. The situation in Singapore and other Asian countries/regions appears worse than in Western countries. Accordingly, the proportion of children not meeting individual MVPA and SV guidelines was relatively high. Moreover, the proportion of children not meeting sleep guidelines was particularly high with an average sleep duration shorter than recommended for the age group. To facilitate better monitoring and surveillance of these behaviours, the adaption of integrated movement guidelines for Singapore and possibly Asia may be advisable. At the same time, considering the common napping behaviours in Asia, it may be necessary to adapt current guidelines in such a way that they include total sleep duration during a 24-h period. Meanwhile, since most interventions have solely targeted individual behaviours, additional strategies to promote movement-related behaviours comprehensively may be particularly important to promote children’s health and tackle growing concerns about childhood obesity. Future interventions should also target maternal behaviours while their children are very young or even during pregnancy.

## Additional file

Additional file 1: **Table S1.** Which of the following activities did your child do *yesterday* and *last weekend*? (If yes, record the times for each activity). **Table S2.** Proportion (%) of children meeting no guidelines, screen viewing (SV), moderate-to-vigorous physical activity (MVPA), sleep duration recommendations and combinations of these recommendations, overall and by socio-demographic and maternal predictors. (DOCX 17 kb).

## Data Availability

The dataset supporting the conclusions of this article can be made available upon request, and after approval by the GUSTO Executive Committee.

## Abbreviations

GUSTO: Growing Up in Singapore Towards healthy Outcomes
MB: Movement behaviour
MVPA: moderate-to-vigorous physical activity
PA: Physical activity
SB: Sedentary behaviour
SV: screen viewing
TV: television viewing
