# Supplementary material for: Socio-demographic and maternal predictors of adherence to 24-hour movement guidelines in Singaporean children
Source: Int J Behav Nutr Phys Act. 2019 Aug 22;16:70. doi: 10.1186/s12966-019-0834-1 (PMC6704617; doi:10.1186/s12966-019-0834-1)
Supplement: Supplementary file 1 — Table S1. Which of the following activities did your child do yesterday and last weekend? (If yes, record the times for each activity). Table S2. Proportion (%) of children meeting no guidelines, screen viewing (SV), moderate-to-vigorous physical activity (MVPA), sleep duration recommendations and combinations of these recommendations, overall and by socio-demographic and maternal predictors. (DOCX 17 kb). [file 12966_2019_834_MOESM1_ESM.docx]

Table S1 Which of the following activities did your child do *yesterday* and *last weekend*? (If yes, record the times for each activity)

| a. Sat or lay still while watching television/DVD/VCR/Blu-ray disc player or other videos on a television screen | | | |
| --- | --- | --- | --- |
| **Yesterday:** □ no □ yes | \|  \|  \| : \|  \|  \|  \| \| --- \| --- \| --- \| --- \| --- \| --- \|   H H M M | | |
| **Saturday:** □ no □ yes | \|  \|  \| : \|  \|  \|  \| \| --- \| --- \| --- \| --- \| --- \| --- \|   H H M M | **Sunday:** □ no □ yes | \|  \|  \| : \|  \|  \|  \| \| --- \| --- \| --- \| --- \| --- \| --- \|   H H M M |
| b. Sat or lay still while watching videos or playing games on a computer, such as a desktop, laptop, or ultrabook | | | |
| **Yesterday:** □ no □ yes | \|  \|  \| : \|  \|  \|  \| \| --- \| --- \| --- \| --- \| --- \| --- \|   H H M M | | |
| **Saturday:** □ no □ yes | \|  \|  \| : \|  \|  \|  \| \| --- \| --- \| --- \| --- \| --- \| --- \|   H H M M | **Sunday:** □ no □ yes | \|  \|  \| : \|  \|  \|  \| \| --- \| --- \| --- \| --- \| --- \| --- \|   H H M M |
| c. Sat or lay still while watching videos or playing games on mobile screen devices, such as a smartphone or tablet | | | |
| **Yesterday:** □ no □ yes | \|  \|  \| : \|  \|  \|  \| \| --- \| --- \| --- \| --- \| --- \| --- \|   H H M M | | |
| **Saturday:** □ no □ yes | \|  \|  \| : \|  \|  \|  \| \| --- \| --- \| --- \| --- \| --- \| --- \|   H H M M | **Sunday:** □ no □ yes | \|  \|  \| : \|  \|  \|  \| \| --- \| --- \| --- \| --- \| --- \| --- \|   H H M M |
| d. Sat or lay still while playing games on video game console, such as Xbox, Nintendo Wii, Nintendo DS, PSP | | | |
| **Yesterday:** □ no □ yes | \|  \|  \| : \|  \|  \|  \| \| --- \| --- \| --- \| --- \| --- \| --- \|   H H M M | | |
| **Saturday:** □ no □ yes | \|  \|  \| : \|  \|  \|  \| \| --- \| --- \| --- \| --- \| --- \| --- \|   H H M M | **Sunday:** □ no □ yes | \|  \|  \| : \|  \|  \|  \| \| --- \| --- \| --- \| --- \| --- \| --- \|   H H M M |

Table S2 Proportion (%) of children meeting no guidelines, screen viewing (SV), moderate-to-vigorous physical activity (MVPA), sleep duration recommendations and combinations of these recommendations, overall and by socio-demographic and maternal predictors.

|  | All | SV | MVPA | Sleep | None |
| --- | --- | --- | --- | --- | --- |
| Overall (N=547) | 5.5 | 70.2 | 59.6 | 13.7 | 11.2 |
| Child sex |  |  |  |  |  |
| Boy | 4.6 | 70.7 | 67.8 | 13.1 | 9.9 |
| Girl | 6.4 | 69.7 | 50.8 | 14.4 | 12.5 |
| Ethnicity |  |  |  |  |  |
| Chinese | 5.0 | 75.9 | 56.7 | 11.9 | 10.3 |
| Indian | 8.3 | 75.0 | 60.4 | 15.6 | 12.5 |
| Malay | 4.5 | 53.0 | 65.9 | 16.7 | 12.1 |
| Birth order |  |  |  |  |  |
| First-born | 3.7 | 73.0 | 56.1 | 11.1 | 11.1 |
| Second-or later-born | 6.9 | 68.0 | 62.4 | 15.8 | 11.2 |
| Household incomes (SGD/month) |  |  |  |  |  |
| <4000 | 5.9 | 59.5 | 56.1 | 15.6 | 13.9 |
| 4000-5999 | 3.4 | 67.5 | 63.2 | 8.5 | 15.4 |
| ≥6000 | 6.3 | 81.3 | 62.5 | 15.0 | 3.8 |
| Maternal education |  |  |  |  |  |
| University | 6.4 | 81.8 | 63.1 | 12.8 | 7.0 |
| Post-secondary | 4.7 | 63.7 | 60.0 | 12.1 | 12.6 |
| Primary or secondary | 5.4 | 64.7 | 55.7 | 16.8 | 14.4 |
| Pre-pregnant weight status |  |  |  |  |  |
| Underweight/normal | 5.6 | 74.7 | 59.4 | 12.1 | 11.5 |
| Overweight | 6.0 | 66.9 | 61.7 | 16.5 | 10.5 |
| Obese | 4.1 | 55.4 | 56.8 | 16.2 | 10.8 |
| Maternal age at delivery |  |  |  |  |  |
| <30 | 4.5 | 65.6 | 65.6 | 12.6 | 11.8 |
| 30-35 | 4.7 | 73.7 | 60.2 | 9.4 | 11.2 |
| ≥ 35 | 7.8 | 67.3 | 54.9 | 20.3 | 10.5 |
| Maternal physical activity before pregnancy |  |  |  |  |  |
| Insufficiently active | 2.0 | 62.6 | 45.5 | 15.2 | 17.2 |
| Sufficiently active | 5.7 | 72.6 | 61.8 | 13.9 | 8.1 |
| Highly active | 7.6 | 69.4 | 64.6 | 13.2 | 13.2 |
| Maternal daily television-viewing time |  |  |  |  |  |
| <2 h | 5.8 | 76.9 | 59.6 | 15.0 | 7.7 |
| 2-3 h | 7.5 | 66.2 | 57.9 | 13.5 | 16.5 |
| ≥ 3 h | 3.4 | 61.2 | 61.2 | 12.2 | 12.2 |
| Maternal daily total sleep time |  |  |  |  |  |
| <7 h | 2.8 | 74.6 | 60.6 | 7.0 | 7.0 |
| 7-9 h | 6.0 | 71.9 | 57.9 | 15.5 | 10.1 |
| ≥ 9 h | 6.0 | 62.7 | 63.4 | 13.4 | 15.7 |
